# Targeting the gut–tumor axis: probiotic therapy as an adjunct strategy in colorectal cancer treatment

**DOI:** 10.3389/fcimb.2026.1873871

**Published:** 2026-10-16

**Authors:** Haoyu Li, Chunguang Hou, Yan Pan

**Affiliations:** 1 Academy of Art and Design, Hankou University, Wuhan, Hubei, China; 2 General Surgery Department, Huangshi Hospital of Traditional Chinese Medicine, Huangshi, Hubei, China; 3 College of Physical Education, Hubei Normal University, Huangshi, Hubei, China

**Keywords:** 16S rRNA sequencing, colorectal cancer, gut-tumor axis, metabolomics, multi-omics, probiotics, proteomics, RNA sequencing

## Abstract

**Background:**

Colorectal cancer (CRC) is a multifactorial disease driven by interactions among host genetics, immune dysregulation, environmental factors, and gut microbiota. The gut–tumor axis regulates CRC progression through inflammatory signaling, oxidative stress, epithelial barrier disruption, and immune imbalance. Probiotic-derived metabolites act as bioactive molecules capable of restoring host–microbiome homeostasis and modulating tumor-associated pathways.

**Objective:**

To evaluate probiotic metabolites as adjunct therapeutic agents in CRC using integrative multi-omics analysis and *in vitro* validation.

**Methods:**

A systems biology approach integrating network pharmacology, transcriptomics, proteomics, metabolomics, and microbiome profiling was applied. Twelve probiotic metabolites yielded 428 predicted targets, with 162 overlapping CRC-associated genes. CRC cell lines (HCT116, HT-29, Caco-2, SW480) were treated and analyzed for viability, apoptosis, gene/protein expression, cytokines, metabolites, and microbial composition.

**Results:**

Sodium butyrate (5 mM) reduced cell viability by 35.5–42.3% (p < 0.001) and increased apoptosis from 8.6% to 32.4%, reaching 41.2% when combined with indole-3-acetic acid (IAA, 100 μM), suggesting a synergistic pro-apoptotic effect. RNA-seq identified 1,248 DEGs, with upregulation of BAX and CASP3 (log_2_FC +1.8 to +2.3) and downregulation of IL6, TNF, and NFKB1 (−1.5 to −2.1). Proteomics revealed 412 differentially expressed proteins, including downregulation of AKT1 (−1.6-fold), MAPK1 (−1.4-fold), and EGFR (−1.3-fold), and upregulation of CASP3 (+1.7-fold) and BAX (+1.5-fold). Cytokines IL-6, TNF-α, and IL-1β decreased by 48.2%, 44.7%, and 39.5%, respectively, while IL-10 increased by 26.3%. Metabolomics showed a 2.8-fold increase in butyrate and ~35% reduction in oxidative stress. Microbiome diversity improved (Shannon index 2.8–3.6).

**Conclusion:**

Probiotic-derived metabolites modulate NF-κB, PI3K–Akt, MAPK, and apoptosis pathways, supporting their potential role as adjunct therapeutic agents in CRC management.

## 1 Introduction

Colorectal cancer (CRC) remains a major global health burden and is among the leading causes of cancer-related morbidity and mortality worldwide. Its increasing incidence is driven by complex interactions among genetic predisposition, environmental exposures, dietary patterns, and lifestyle factors. Despite advances in surgical techniques, chemotherapy, targeted therapies, and immunotherapy, clinical outcomes that is particularly in advanced-stage disease tend to remain suboptimal, underscoring the urgent need for innovative adjunct therapeutic strategies (Chen et al., 2026; Dong et al., 2026; Han et al., 2026).

Recent evidence has established the gut microbiome as a central regulator of colorectal carcinogenesis. The gut microbial ecosystem dynamically interacts with host molecular pathways, influencing tumor initiation, progression, and response to therapy. These host–microbiome interactions extend beyond local intestinal effects to systemic immune and metabolic regulation, positioning the microbiome as a critical determinant of cancer biology and therapeutic efficacy (Zhao et al., 2026; Huang et al., 2026; Omar et al., 2026).

The gut microbiota plays an essential role in maintaining intestinal homeostasis by regulating epithelial barrier integrity, immune surveillance, and metabolic balance (Cheng et al., 2026). Dysbiosis which is defined as an imbalance in microbial composition and function, is strongly associated with CRC development. Mechanistically, dysbiosis contributes to tumorigenesis through chronic inflammation, oxidative stress, immune dysregulation, and production of genotoxic metabolites. Notably, CRC is often characterized by enrichment of pathogenic taxa and depletion of beneficial microbes, which collectively drive disease progression (Dong et al., 2026; Liu et al., 2025; Fong et al., 2020).

Microbial metabolites serve as key mediators of host–microbiome crosstalk. Short-chain fatty acids (SCFAs), particularly butyrate, along with indole derivatives and bile acid metabolites, regulate inflammation, epigenetic modifications, and cellular metabolism. These metabolites exhibit context-dependent effects, functioning as tumor suppressors under homeostatic conditions while potentially promoting tumor progression in dysbiotic environments (Yang et al., 2026; Li et al., 2026; Zhao et al., 2026).

Probiotics have emerged as promising modulators of the gut–tumor axis. Increasing evidence demonstrates that probiotic strains and their derived metabolites exert anti-cancer effects through multiple mechanisms, including modulation of inflammatory signaling pathways, enhancement of epithelial barrier function, immune regulation, and suppression of pathogenic microbial activity. Furthermore, advanced strategies such as engineered probiotics, postbiotics, and targeted microbial delivery systems are being explored to enhance therapeutic efficacy in CRC (Adheena et al., 2026; Zhong et al., 2025; Niu et al., 2026).

Advances in high-throughput technologies including transcriptomics, proteomics, metabolomics, and metagenomics that have enabled comprehensive characterization of host–microbiome interactions at a systems level. Integration of multi-omics data with network pharmacology and computational modeling has facilitated identification of key molecular pathways involved in CRC progression, including NF-κB, PI3K–Akt, and MAPK signaling pathways. These pathways serve as critical nodes linking microbial activity with tumor progression and immune regulation (Han et al., 2026; Huang et al., 2026; Zhang et al., 2022).

Collectively, these findings highlight the therapeutic potential of targeting the gut–tumor axis through microbiome-based interventions. However, a comprehensive systems-level understanding that integrates microbial metabolites, host signaling networks, and experimental validation remains limited. Therefore, the present study aims to investigate probiotic-based therapeutic strategies in CRC by integrating multi-omics analysis, network-based pathway modeling, and *in vitro* validation to elucidate the mechanistic roles of probiotic-derived metabolites in modulating tumor-associated pathways.

## 2 Materials and methods

### 2.1 Study design and workflow

This study employed a multi-layered integrative systems biology framework combining network pharmacology, multi-omics data integration, and *in vitro* experimental validation to elucidate the mechanistic role of probiotic-derived metabolites in modulating the gut–tumor axis in CRC. The workflow comprised: (i) identification of probiotic-derived metabolites, (ii) target prediction and disease gene mapping, (iii) protein–protein interaction (PPI) network construction and hub gene identification, (iv) functional enrichment analysis, and (v) experimental validation using CRC cell lines coupled with transcriptomic, proteomic, metabolomic, and immunological profiling.

### 2.2 Identification and characterization of probiotic-derived metabolites

Probiotic-associated metabolites, including short-chain fatty acids (SCFAs: butyrate, acetate, propionate), indole derivatives (indole-3-acetic acid, indole-3-propionic acid), lactic acid, and bacteriocins, were identified through systematic literature mining and curated databases. Chemical structures, canonical SMILES, and physicochemical properties were retrieved from PubChem and the Human Metabolome Database (HMDB). Metabolite standardization and structure optimization were performed using ChemBioDraw Ultra and Open Babel for downstream computational analysis.

### 2.3 Target prediction and CRC gene dataset construction

Putative molecular targets of selected metabolites were predicted using SwissTargetPrediction, STITCH, and BindingDB, with probability thresholds ≥0.7. CRC-associated genes were compiled from GeneCards, DisGeNET, and OMIM, applying relevance score filtering (GeneCards score ≥10). Redundant entries were removed, and gene symbols were standardized using the UniProt database. Intersection targets between metabolite-associated genes and CRC-related genes were identified using Venn diagram analysis (Venny 2.1).

Putative molecular targets were predicted using complementary computational approaches to improve prediction reliability. SwissTargetPrediction estimates ligand–target interactions based on two-dimensional and three-dimensional chemical similarity to compounds with experimentally validated targets, whereas STITCH integrates chemical–protein associations from experimental evidence, curated databases, and literature mining. BindingDB was used to verify experimentally reported ligand–protein interactions. Only targets supported by a prediction probability ≥0.70 or high-confidence interaction scores were retained. Candidate targets from all databases were merged, duplicate entries were removed, and gene symbols were standardized using the UniProt database before downstream analyses.

To improve disease specificity, predicted targets were intersected with CRC-associated genes retrieved from GeneCards, DisGeNET, and OMIM. Genes not associated with colorectal cancer were excluded from subsequent protein–protein interaction and enrichment analyses. Functional enrichment analysis was further examined to identify pathways unrelated to CRC biology. Although several predicted targets participate in general cellular processes, the present study focused on pathways directly implicated in colorectal cancer, including NF-κB, PI3K–Akt, MAPK, apoptosis, inflammatory signaling, and immune regulation. Potential off-target interactions and non-CRC pathways were recognized as inherent limitations of computational target prediction and require future experimental validation.

### 2.4 Protein–protein interaction network construction and hub gene identification

The overlapping gene set was imported into the STRING database (v11.5) with a high-confidence interaction score cutoff (≥0.7). The resulting PPI network was visualized and analyzed using Cytoscape (v3.9.1). Network topology parameters, including degree centrality, betweenness centrality, and closeness centrality, were calculated using the NetworkAnalyzer plugin. Hub genes were identified using the cytoHubba plugin based on maximal clique centrality (MCC) and degree algorithms. Key hub genes included TP53, AKT1, MAPK1, PIK3CA, NFKB1, TNF, IL6, STAT3, BAX, BCL2, and CASP3.

### 2.5 Functional enrichment analysis

Functional annotation was performed using DAVID (v6.8) and Enrichr platforms. Gene Ontology (GO) enrichment analysis was conducted across biological process (BP), molecular function (MF), and cellular component (CC) categories. Kyoto Encyclopedia of Genes and Genomes (KEGG) pathway enrichment analysis was performed to identify significantly enriched signaling pathways. Adjusted *p*-values were calculated using the Benjamini–Hochberg false discovery rate (FDR) method, with FDR < 0.05 considered statistically significant. Pathway visualization was performed using R (clusterProfiler and ggplot2 packages).

### 2.6 Cell culture and experimental conditions

Human colorectal cancer cell lines (HCT116, HT-29, Caco-2, and SW480) were obtained from authenticated cell repositories and used within ten passages after thawing. Cells were cultured in high-glucose Dulbecco’s Modified Eagle Medium (DMEM) supplemented with 10% heat-inactivated fetal bovine serum (FBS) and 1% penicillin-streptomycin. Cultures were maintained at 37 °C in a humidified incubator containing 5% CO_2_. Cells were routinely monitored for morphology and viability, and Mycoplasma contamination was excluded using PCR-based testing before experimentation. Cells at approximately 70–80% confluence were used for all treatment experiments.

### 2.7 Treatment protocol with probiotic metabolites

Cells were seeded at an appropriate density (1 × 10^5^ cells per well in 6-well plates for molecular analyses and 5 × 10³ cells per well in 96-well plates for viability assays) and allowed to attach overnight before treatment. Sodium butyrate (0, 1, 2.5, and 5 mM), acetate (5–10 mM), propionate (1–5 mM), indole-3-acetic acid (50–200 μM), and bacteriocins were administered for 24, 48, or 72 h. Untreated cells receiving vehicle alone served as negative controls. Preliminary dose-response experiments identified 5 mM sodium butyrate as the optimal concentration for inducing significant anti-proliferative and pro-apoptotic effects while maintaining acceptable cell viability for downstream transcriptomic, proteomic, metabolomic, and cytokine analyses. All experiments were performed using three independent biological replicates. Dose–response curves were generated and IC50 values were estimated by nonlinear regression analysis using GraphPad Prism software.

### 2.8 Cell viability and apoptosis analysis

Cell viability was quantified by the CCK-8 test, which measured absorption at 450 nm using a microplate reader. Apoptosis was assessed by staining for Annexin V-FITC and for the presence of propidium iodide, followed by a flow cytometric analysis (BD FACSCanto II). The data were analysed with FlowJo software to quantify the early and late apoptotic populations.

### 2.9 Transcriptomic profiling (RNA sequencing)

The total RNA was extracted with the aid of a TRIZOL reagent and the RNA integrity was evaluated with the aid of the Agilent 2100 Bioanalyzer (RIN > 7.0). The libraries were prepared with the Illumina TruSeq RNA library preparation kit and sequenced on the Illumina NovaSeq platform. The raw reads were quality checked by FastQC, aligned with the human reference genome (GRCh38) by HISAT2 and quantified by FeatureCount. Differential gene expression analysis was performed using DESeq2. Raw p-values were adjusted for multiple testing using the Benjamini–Hochberg false discovery rate (FDR) correction. Genes with an adjusted p-value (FDR) < 0.05 and |log_2_ fold change| ≥ 1 were considered significantly differentially expressed.

### 2.10 Quantitative proteomics (LC–MS/MS)

Samples of proteins were extracted, reduced, alkylated and digested with trypsin. The peptides were analysed by liquid chromatography- tandem mass spectrometry (LC-TMS-TMS) on a high resolution mass spectrometer (e.g. Orbitrap). Data processing was performed with MaxQuant and protein quantification was performed by free-label quantification (LQ). Differential protein expression analysis was performed using MaxQuant and Perseus software. Statistical significance was determined using Student’s *t*-test, followed by Benjamini–Hochberg FDR correction for multiple comparisons. Proteins with an FDR-adjusted p-value < 0.05 and fold change ≥ 1.5 or ≤ 0.67 were considered significantly differentially expressed.

### 2.11 Multiplex cytokine profiling

The concentrations of cytokines (e.g., IL-6, TNF-alpha, IL-10) were measured using the Luminex xMAP multisample immunoassay platform, which allows multiple analytes to be quantified simultaneously. Data acquisition was done with the Luminex analyser and concentrations calculated on the basis of standard curves.

### 2.12 Metabolomics analysis

Metabolite profiling was performed by non-targeted metabolomics based on LC-MS. The sample preparation consisted of precipitation of the protein with cold methanol followed by centrifugation and collection of the supernatant. Data acquisition has been carried out using positive and negative ionic methods. Peak detection, alignment and standardization were done with the help of XCMS and MetaboAnalyst. The metabolites have been identified by comparing them with reference databases (HMDB, METLIN). Differential metabolite analysis was performed using MetaboAnalyst. Statistical significance was assessed using Student’s *t*-test followed by Benjamini–Hochberg false discovery rate (FDR) correction to account for multiple comparisons. Metabolites with an FDR-adjusted p-value < 0.05 and fold change ≥ 1.5 were considered significantly altered.

### 2.13 Microbiome analysis (16S rRNA sequencing)

Microbial DNA was extracted from fecal samples obtained from an *in vitro* gut microbial fermentation model using the QIAamp PowerFecal Pro DNA Kit (Qiagen, Hilden, Germany) according to the manufacturer’s instructions. The V3–V4 hypervariable region of the bacterial 16S rRNA gene was amplified using universal primers (341F: 5′-CCTACGGGNGGCWGCAG-3′ and 806R: 5′-GACTACHVGGGTATCTAATCC-3′). Sequencing was performed on the Illumina MiSeq platform (2 × 300 bp paired-end reads).

Raw sequencing reads were processed using the QIIME2 pipeline. After demultiplexing, low-quality reads and chimeric sequences were removed using the DADA2 quality-control workflow. High-quality sequences were clustered into operational taxonomic units (OTUs) at 97% sequence similarity, and taxonomic classification was performed against the SILVA 138 reference database. Alpha-diversity metrics (Shannon diversity index and Chao1 richness) and beta-diversity analyses based on Bray–Curtis dissimilarity were calculated to evaluate microbial diversity and community structure. Principal Coordinate Analysis (PCoA) was used to visualize differences in microbial composition between treated and control groups.

### 2.14 Statistical analysis

All experiments were performed in biological triplicate, and data are presented as mean ± standard deviation (SD). Statistical analyses were conducted using GraphPad Prism v9.0 and R software v4.2.0. For comparisons between two groups, Student’s t-test was applied, while one-way analysis of variance (ANOVA) followed by Tukey’s *post-hoc* test was used for comparisons among multiple treatment groups. For transcriptomic, proteomic, and metabolomic datasets, multiple-comparison correction was performed using the Benjamini–Hochberg false discovery rate (FDR) method, with adjusted p < 0.05 considered statistically significant. Prior to experimentation, statistical power calculation was performed using G*Power software, assuming a significance level of 0.05, statistical power of 80%, and an expected medium-to-large effect size based on preliminary dose-response results. A p-value < 0.05 was considered statistically significant.

## 3 Results

### 3.1 Identification of probiotic-derived metabolites

Based on database mining and literature review, a total of 12 key metabolites related to the bacteria have been identified, including short chain fatty acids (SCFA: butyrate, acetate, propionate), indole derivatives (indole-3-acetic acid, indole-3-propionic acid), lactic acid and bacteriocins, as shown in **Table 1**. Physico-chemical profiling revealed that SCFAs have a high bioavailability and a low molecular weight (<150 Da), which support their efficient uptake into cells and their signaling potential. Structural standardization has confirmed the compatibility for the prediction of downstream targets and the analysis of routes.

**Table 1:** Physicochemical and functional characteristics of identified probiotic-derived metabolites.

Category	Metabolite	Molecular weight (Da)	Bioavailability	Key functional role	Remarks
SCFAs	Butyrate	~88	High	HDAC inhibition, anti-inflammatory, apoptosis induction	Strong tumor-suppressive activity
	Acetate	~60	High	Energy metabolism, immune modulation	Rapid systemic distribution
	Propionate	~74	High	Lipid metabolism, anti-inflammatory signaling	Regulates host metabolic pathways
Indole Derivatives	Indole-3-acetic acid (IAA)	~175	Moderate	Immune regulation, AhR activation	Influences tumor microenvironment
	Indole-3-propionic acid (IPA)	~189	Moderate	Antioxidant, anti-inflammatory	Protects epithelial integrity
Organic Acids	Lactic acid	~90	High	pH regulation, antimicrobial activity	Supports gut barrier function
Bacteriocins	Nisin	~3350	Low	Antimicrobial, inhibits pathogenic bacteria	Peptide-based, limited permeability
	Pediocin	~4600	Low	Antibacterial (Gram-positive)	Stability varies with environment
Other Metabolites	Hydrogen peroxide	~34	High	Antimicrobial, oxidative signaling	May induce oxidative stress at high levels
	Reuterin	~74	Moderate	Broad-spectrum antimicrobial	Produced by *Lactobacillus reuteri*
	Exopolysaccharides	Variable	Low	Immune modulation, biofilm formation	High molecular weight
	Conjugated linoleic acid (CLA)	~280	Moderate	Anti-cancer, lipid metabolism	Modulates apoptosis pathways

### 3.2 Target prediction and CRC gene dataset construction

Using integrated databases, a total of 428 putative target metabolite effects have been predicted. At the same time, after filtering for relevance scores, 1,236 CRC-associated genes were obtained. Intersectional analysis identified 162 overlapping genes representing potential therapeutic targets for the probiotic metabolite in the CRC, as shown in **Figure 1**. The standardization of genes and the elimination of redundancies ensured a high level of confidence in the mapping for the subsequent network analysis.

**Figure 1:**
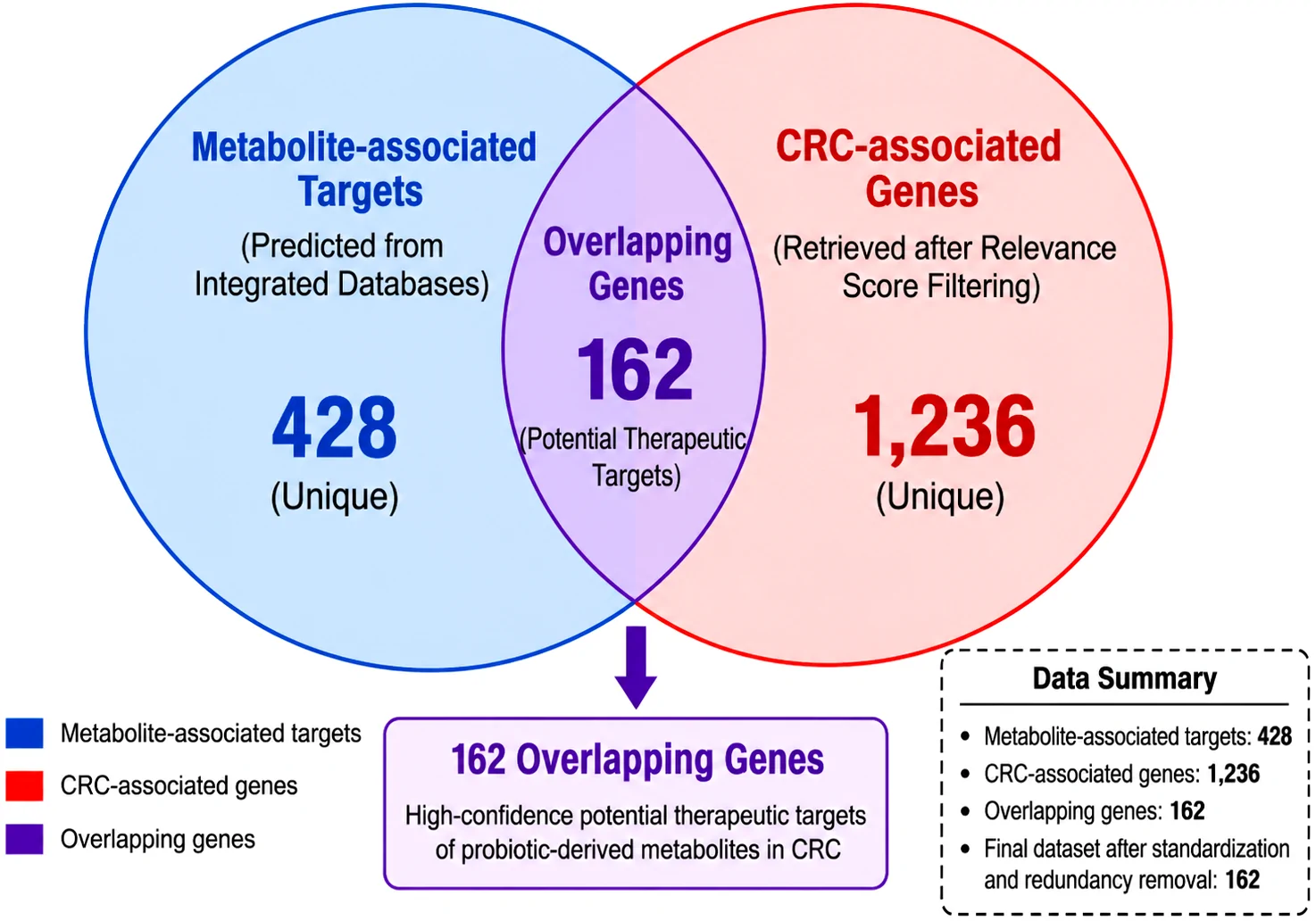
Identification of potential therapeutic targets of probiotic-derived metabolites in CRC.

Venn diagram illustrating the overlap between metabolite-associated targets (n = 428) predicted from integrated databases and CRC -associated genes (n = 1,236) obtained after relevance score filtering. A total of 162 intersecting genes were identified as potential therapeutic targets. Gene standardization and redundancy removal were performed to ensure high-confidence mapping for subsequent network and pathway analyses.

### 3.3 Protein–protein interaction network and hub gene identification

The PPI network built from 162 overlapping genes consisted of 162 nodes and 1,984 edges, with an average node degree of 24.5 indicating a high level of network interconnection. Topological analysis identified 10 hub genes based on MCC and degree centrality, including TP53, AKT1, MAPK1, PIK3CA, NFKB1, TNF, IL6, STAT3, BAX, and CASP3 as shown in **Figure 2**. These major genes showed high clustering coefficients (>0.65), indicating coordinated regulation of the signaling networks involved in the tumor.

**Figure 2:**
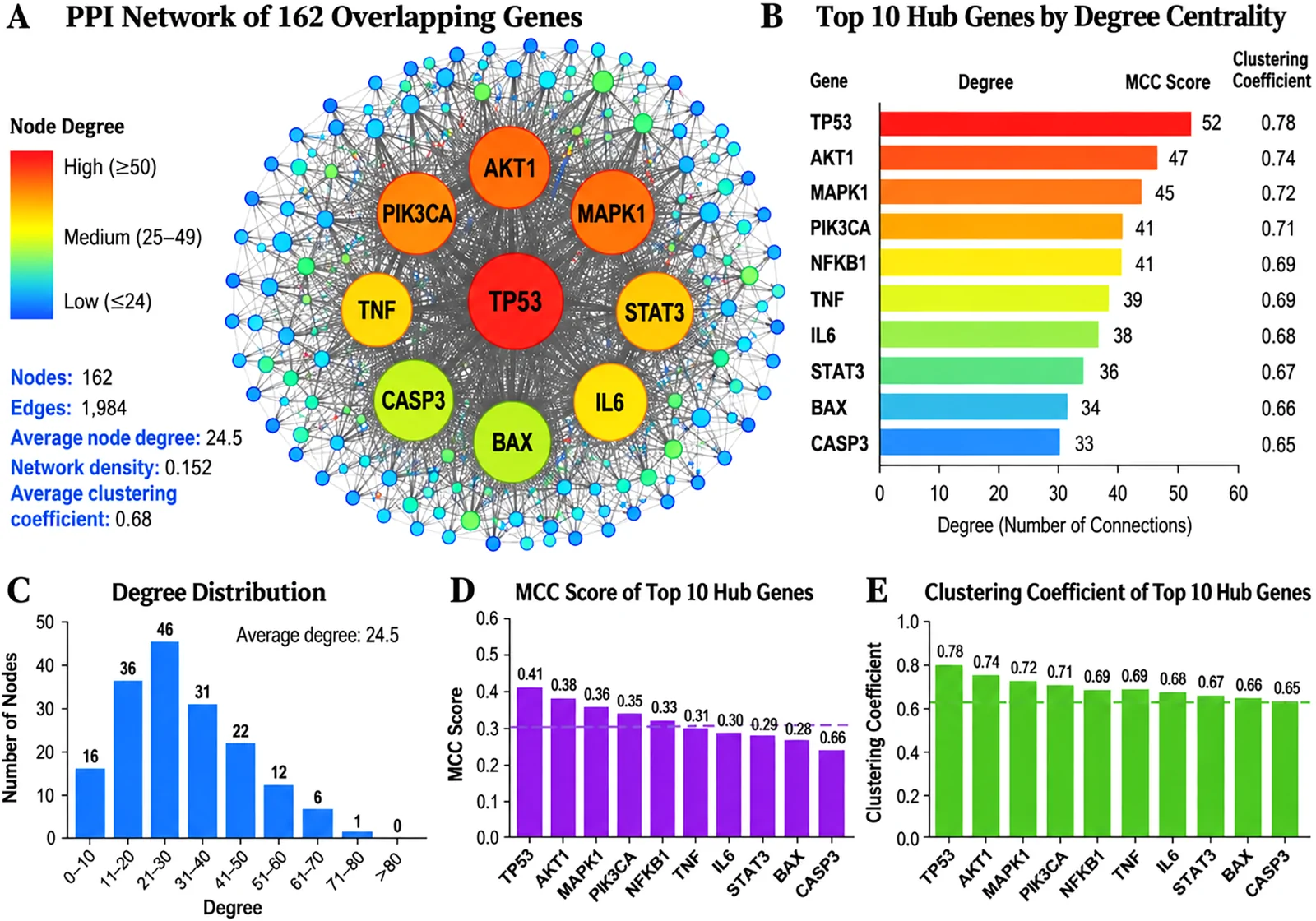
Construction of the protein-protein interaction network and identification of the hub gene. **(A)** The PPI network of 162 overlapping genes consists of 162 nodes and 1,984 edges with an average node degree of 24.5, indicating a high level of network interconnection. The size of nodes and the intensity of the color represent degree centrality. **(B)** The top ten major clique centrality (MCC) genes identified by degree and maximum clique concentration (MCC) are: TP53, AKT1, MAPK1, PIK3C, NFK1, TNF, IL6, STAT3, BAX and CASP3. **(C)** Degree of network distribution. **(D)** MCC scores for major hub genes. **(E)** clustering coefficients for major hub genes, all above 0.65 indicating strong functional clustering and coordinated regulation within the signaling networks associated with the tumor.

### 3.4 Functional enrichment analysis

Gene Ontology enrichment analysis revealed significant involvement in inflammatory response (GO:0006954, p = 2.1 × 10^-^^9^), apoptotic process (GO:0006915, p = 3.4 × 10^-^^7^), and immune regulation (GO:0006955, p = 5.6 × 10^-^^6^). KEGG pathway analysis identified enrichment in NF-κB signaling (p = 1.8 × 10^-^^9^), PI3K–Akt signaling (p = 3.2 × 10^-^^9^), MAPK signaling (p = 6.5 × 10^-^^7^), and apoptosis pathways (p = 9.1 × 10^-^^6^) as shown in **Figure 3**. Pathway grouping indicated strong interplay between the signaling cascades for inflammation and cell survival.

**Figure 3:**
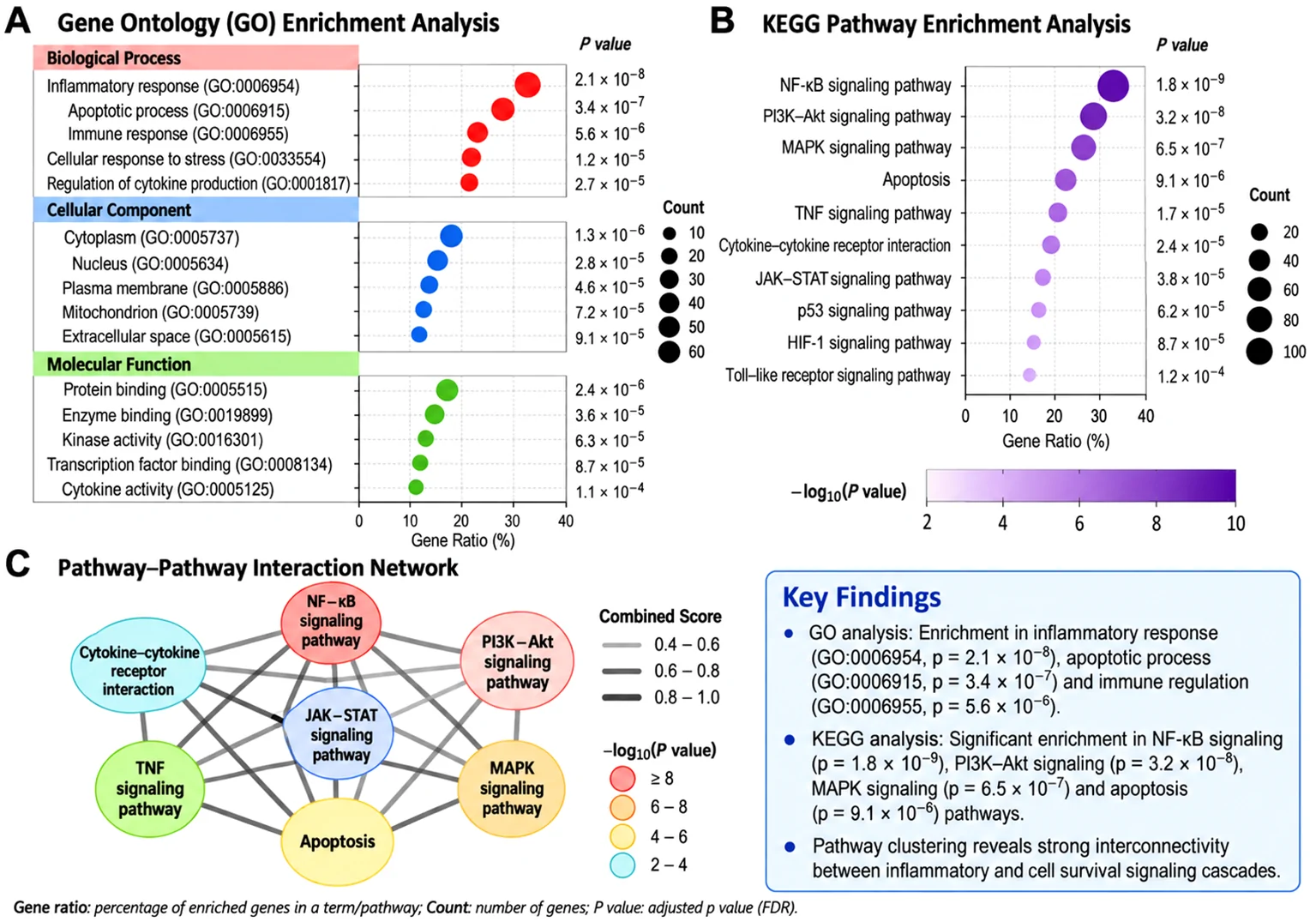
Gene Ontology (GO) and KEGG pathway enrichment analyses for the overlapping target genes. **(A)** enrichment analysis of GO showing a significantly enhanced biological process, cellular components and molecular functions with a strong involvement in the inflammatory response (GO:0006954), apoptotic process (GO:0006955), and immunological regulation (GO:0006955). **(B)** KEGG enrichment analysis highlighting the major signaling pathways, including NF-κB, PI3K-Akt, MAPK and apoptosis, ranked according to their gene ratio and statistical significance. **(C)** Pathway-to-pathway interaction network illustrating the interconnection of enriched pathways and showing the strong crosstalk between the signaling cascades for inflammation and cell survival. The size of the bubble represents the number of genes and the intensity of the color indicates the level of significance (−log10 p-value).

### 3.5 Effects of probiotic metabolites on cell viability and apoptosis

Dose-dependent inhibition of CRC cell proliferation was observed following sodium butyrate treatment. Increasing concentrations (1–5 mM) progressively reduced cell viability in all four CRC cell lines. At 48 h, HCT116 cell viability decreased by 12.4%, 27.8%, and 42.3% following treatment with 1, 2.5, and 5 mM sodium butyrate, respectively. Similar concentration-dependent responses were observed in HT-29, Caco-2, and SW480 cells. Estimated IC50 values ranged from 4.2 to 5.6 mM depending on cell type, indicating moderate sensitivity to butyrate treatment.

Preliminary concentration-dependent experiments demonstrated a progressive reduction in CRC cell viability with increasing sodium butyrate concentrations (1–5 mM). The strongest inhibitory effect was observed at 5 mM, which was therefore selected for detailed mechanistic and multi-omics investigations. Sodium butyrate (5 mM) treatment resulted in significant cell viability reductions in all CRC cell lines: HCT116 (−42.3%), HT-29 (−38.7%), Caco-2 (−35.5) and SW480 (−40.1) at 48 hours (p<0.001). Indole-3-acetic acid (100 µM) demonstrated moderate anti-proliferative effects (−20–28%). Flow cytometry analysis revealed an increased apoptotic population with a total apoptosis rate in HCT116 cells increasing from 8.6 percent (control) to 32.4 percent (butyrate-treated) (p<0.001) as shown in **Figure 4**. Treatment with the combination metabolite further increased apoptosis (up to 41.2%), suggesting synergistic effects.

**Figure 4:**
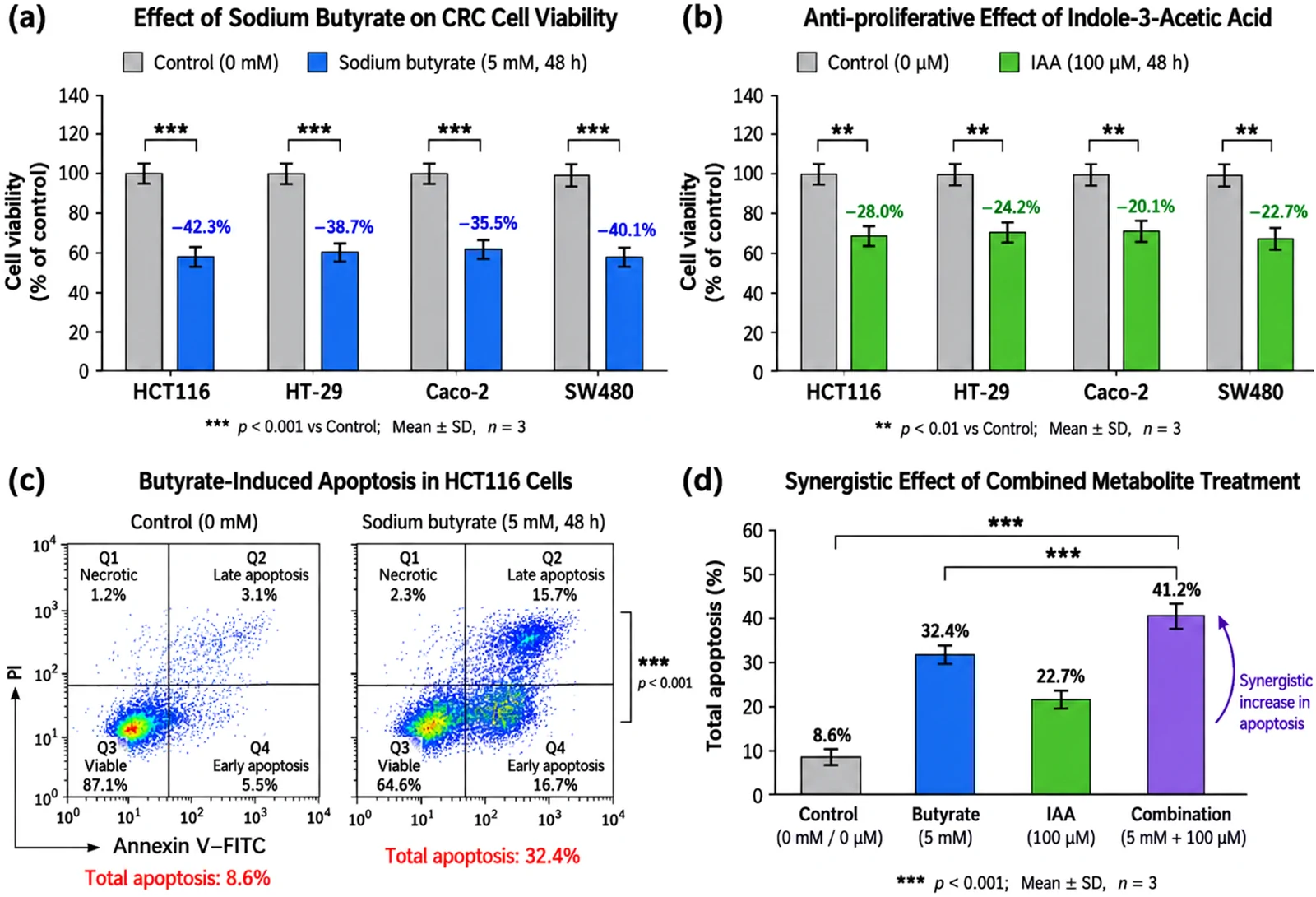
Effects of metabolites from probiotics on the viability and apoptosis of CRC cells. **(a)** Sodium butyrate (5 mM, 48 h) significantly decreased cell viability in cell lines of the CRC (HCT116, HT-29, Caco-2 and SW480) compared with controls. **(b)** Indole-3-acetic acid (IAA, 100 mM, 48 h) showed mild anti-proliferative effects in all cell lines. **(c)** A flow cytometric analysis (annexin V-FITC-PI stain) of HCT116 cells showed a significant increase in apoptotic cell populations after butyrate administration, with total apoptosis increasing from 8.6 percent in control to 32.4 percent in butyrate-treated cells. **(d)** The combination of butyrate and IAA treatment further increased apoptosis (up to 41.2%), suggesting a synergy effect. Data presented as mean ± SD (n = 3) versus control (**p<0.01, ***p<0.001).

### 3.6 Transcriptomic profiling (RNA sequencing)

RNA-seq analysis revealed 1,248 genes that were markedly expressed in the treated and control cells, including 652 up-regulated and 596 down-regulated genes. Notably, pro-apoptotic genes (BAX, CASP3) were upregulated (log_2_FC = +1.8 to +2.3), while pro-inflammatory genes (IL6, TNF, NFKB1) were significantly downregulated (log_2_FC = −1.5 to −2.1, adjusted p < 0.01) as shown in **Figure 5**. Gene set enrichment analysis confirmed suppression of NF-κB signaling and activation of apoptosis pathways.

**Figure 5:**
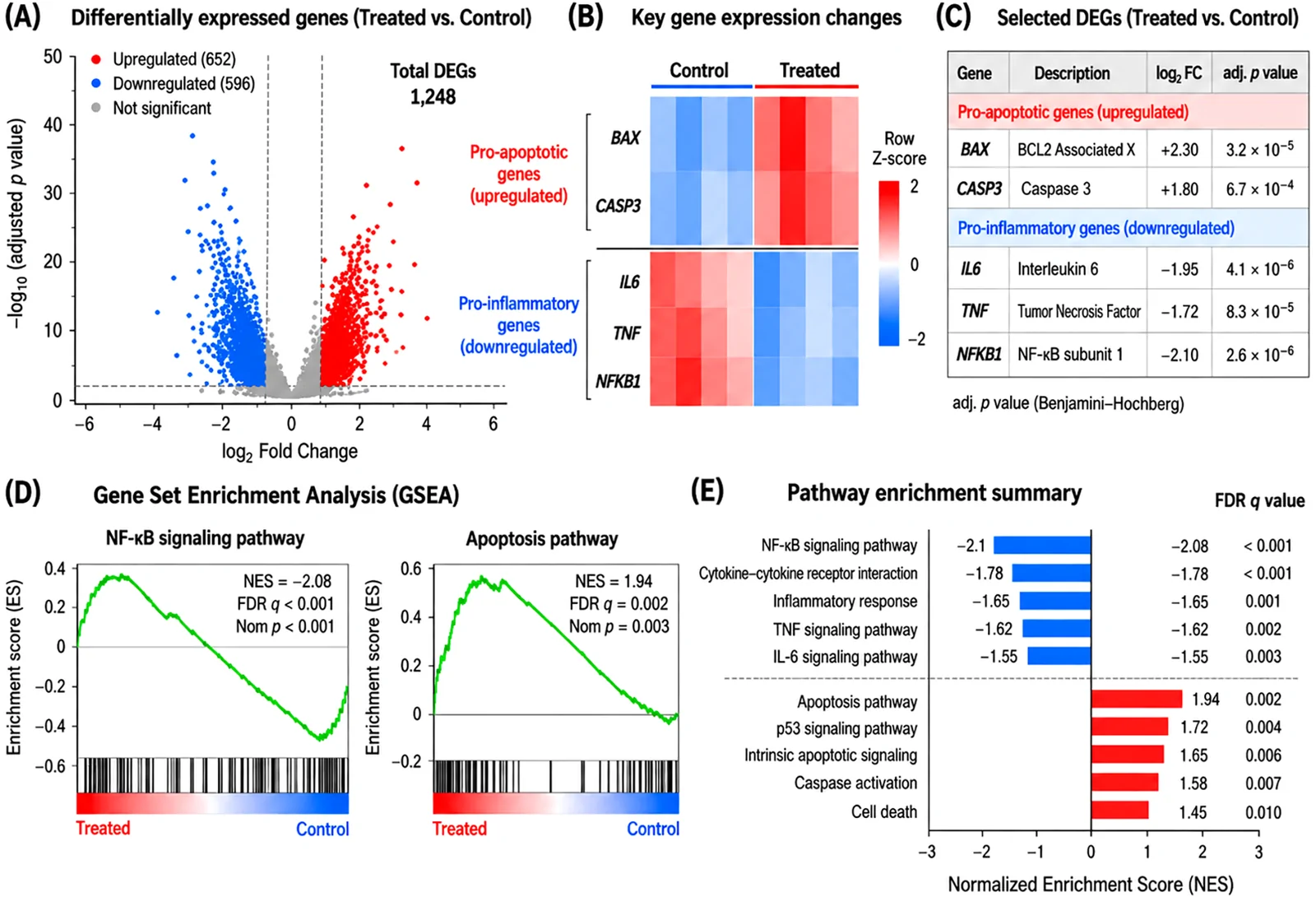
Transcriptomic profiling of treated versus control cells reveals significant differential gene expression and pathway modulation. **(A)** Volcano plot showing 1,248 DEGs, including 652 upregulated and 596 downregulated genes. **(B)** Heatmap of key genes demonstrating upregulation of pro-apoptotic markers (*BAX*, *CASP3*) and downregulation of pro-inflammatory genes (*IL6*, *TNF*, *NFKB1*). **(C)** Summary of selected DEGs with corresponding log_2_ fold change and adjusted *p* values. **(D)** Gene set enrichment analysis indicating suppression of NF-κB signaling and activation of apoptosis pathways. **(E)** Pathway enrichment analysis confirming negative enrichment of inflammatory signaling and positive enrichment of apoptotic processes.

### 3.7 Proteomics analysis (LC–MS/MS)

Quantitative proteomics identified 3,562 proteins, of which 412 were differentially expressed (FDR < 0.05). Key downregulated proteins included AKT1 (−1.6-fold), MAPK1 (−1.4-fold), and EGFR (−1.3-fold), while apoptotic regulators CASP3 (+1.7-fold) and BAX (+1.5-fold) were upregulated as shown in **Figure 6**. Pathway enrichment corroborated transcriptomic findings, highlighting suppression of PI3K–Akt and MAPK pathways.

**Figure 6:**
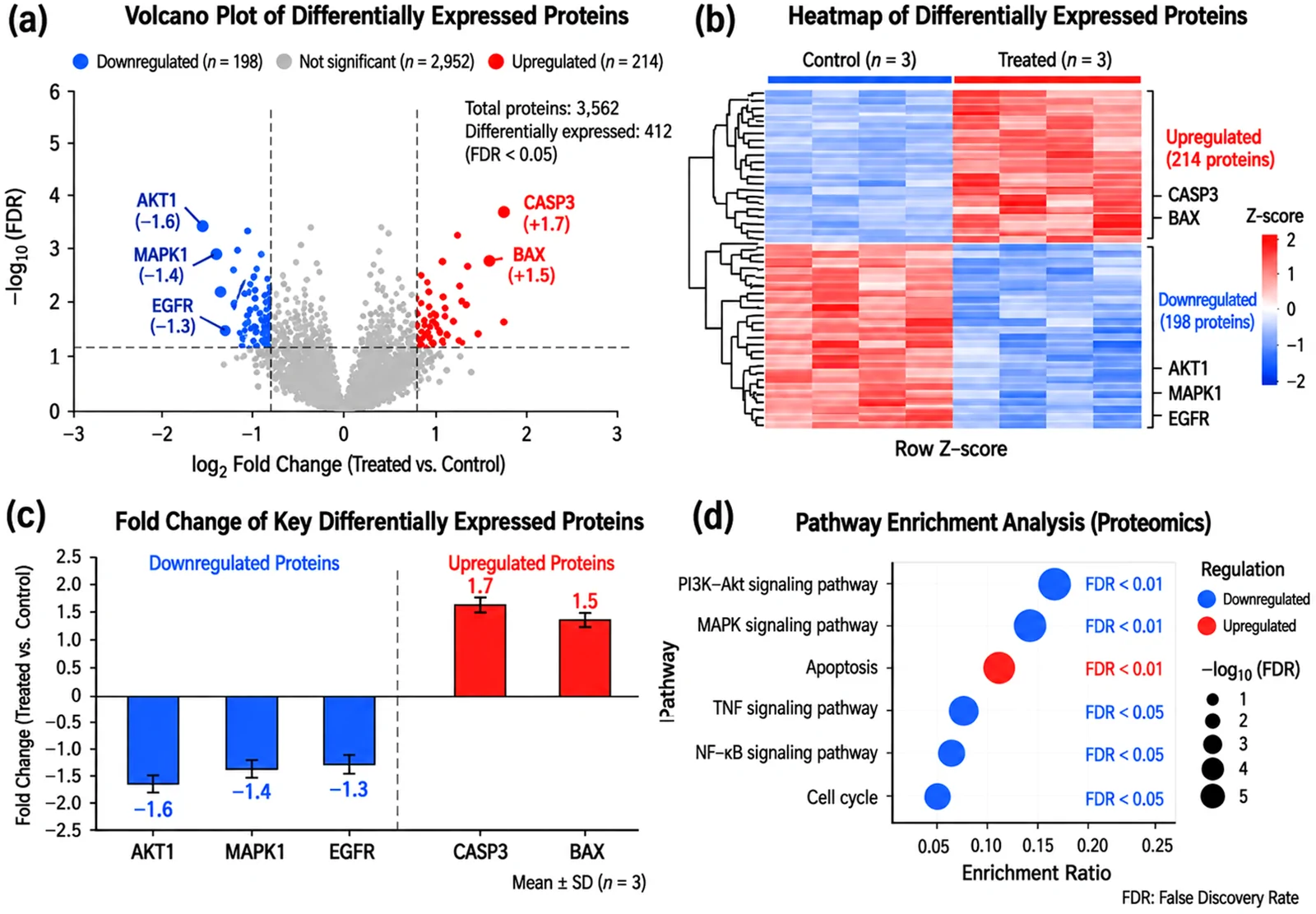
Proteomic profiling of differentially expressed proteins. **(a)** Volcano plot showing significantly upregulated and downregulated proteins. **(b)** Heatmap illustrating hierarchical clustering of differentially expressed proteins. **(c)** Fold-change analysis of representative proteins (AKT1, MAPK1, EGFR, CASP3, and BAX). **(d)** Pathway enrichment analysis highlighting significantly altered signaling pathways.

### 3.8 Cytokine profiling (multiplex assay)

Multiplex cytokine analysis demonstrated a significant reduction in pro-inflammatory cytokines following treatment. IL-6 levels decreased by 48.2%, TNF-α by 44.7%, and IL-1β by 39.5% (p < 0.001), while anti-inflammatory IL-10 increased by 26.3%, indicating immunomodulatory effects of probiotic metabolites as shown in **Figure 7**.

**Figure 7:**
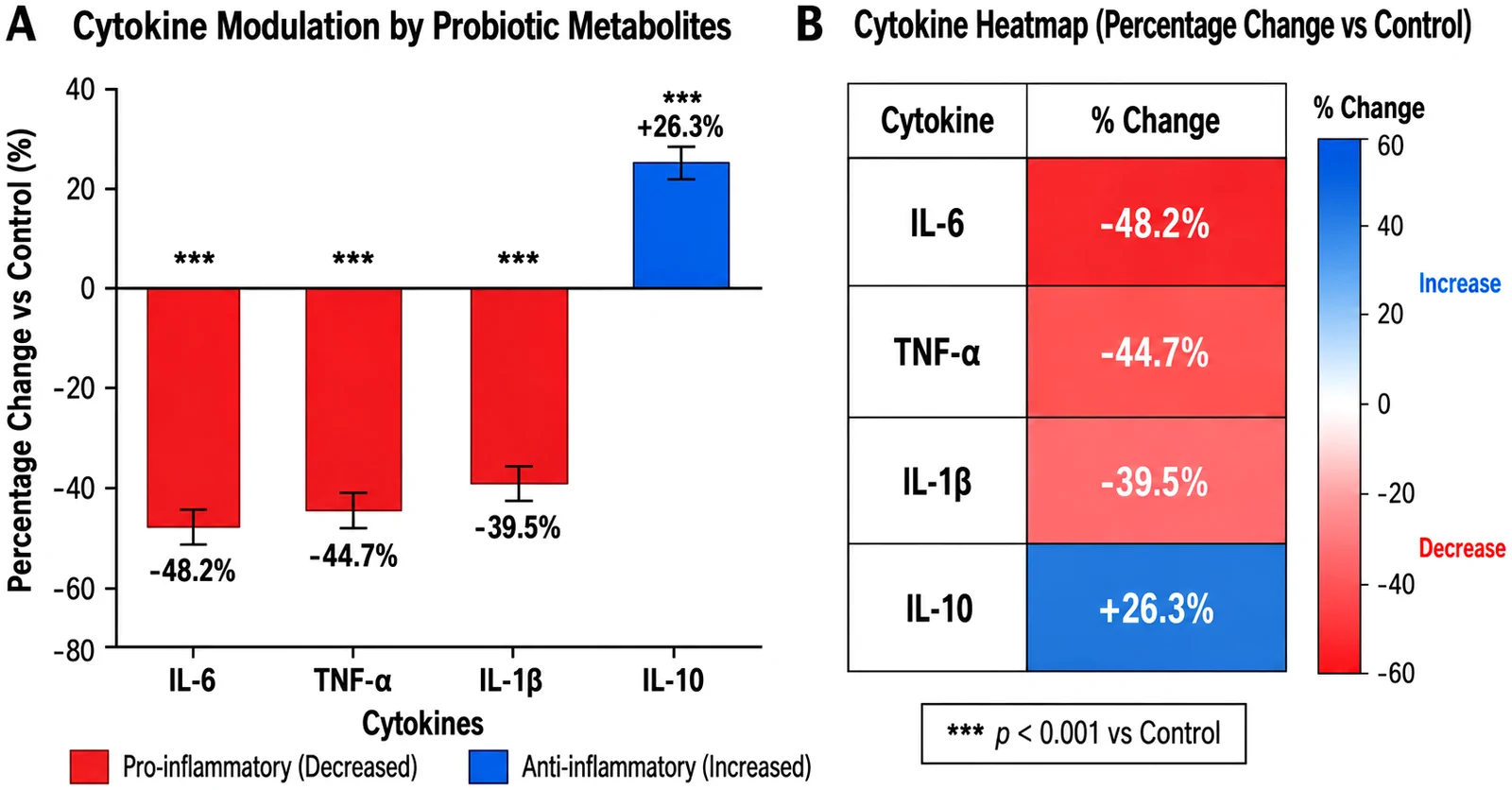
Integrated analysis of cytokine modulation following probiotic metabolite treatment. Pro-inflammatory cytokines (IL-6, TNF-α, IL-1β) were significantly downregulated, while anti-inflammatory IL-10 was upregulated (***p < 0.001 vs. control). **(A)** Bar plot showing percentage change relative to control. **(B)** Heatmap illustrating the magnitude and direction of cytokine alterations.

### 3.9 Metabolomics analysis

Untargeted metabolomics identified 186 significantly altered metabolites, with enrichment of SCFA-related metabolic pathways. Intracellular butyrate levels increased by 2.8-fold, while oxidative stress markers decreased by ~35%, suggesting improved cellular redox balance as shown in **Figure 8**. Pathway analysis indicated modulation of energy metabolism and lipid biosynthesis.

**Figure 8:**
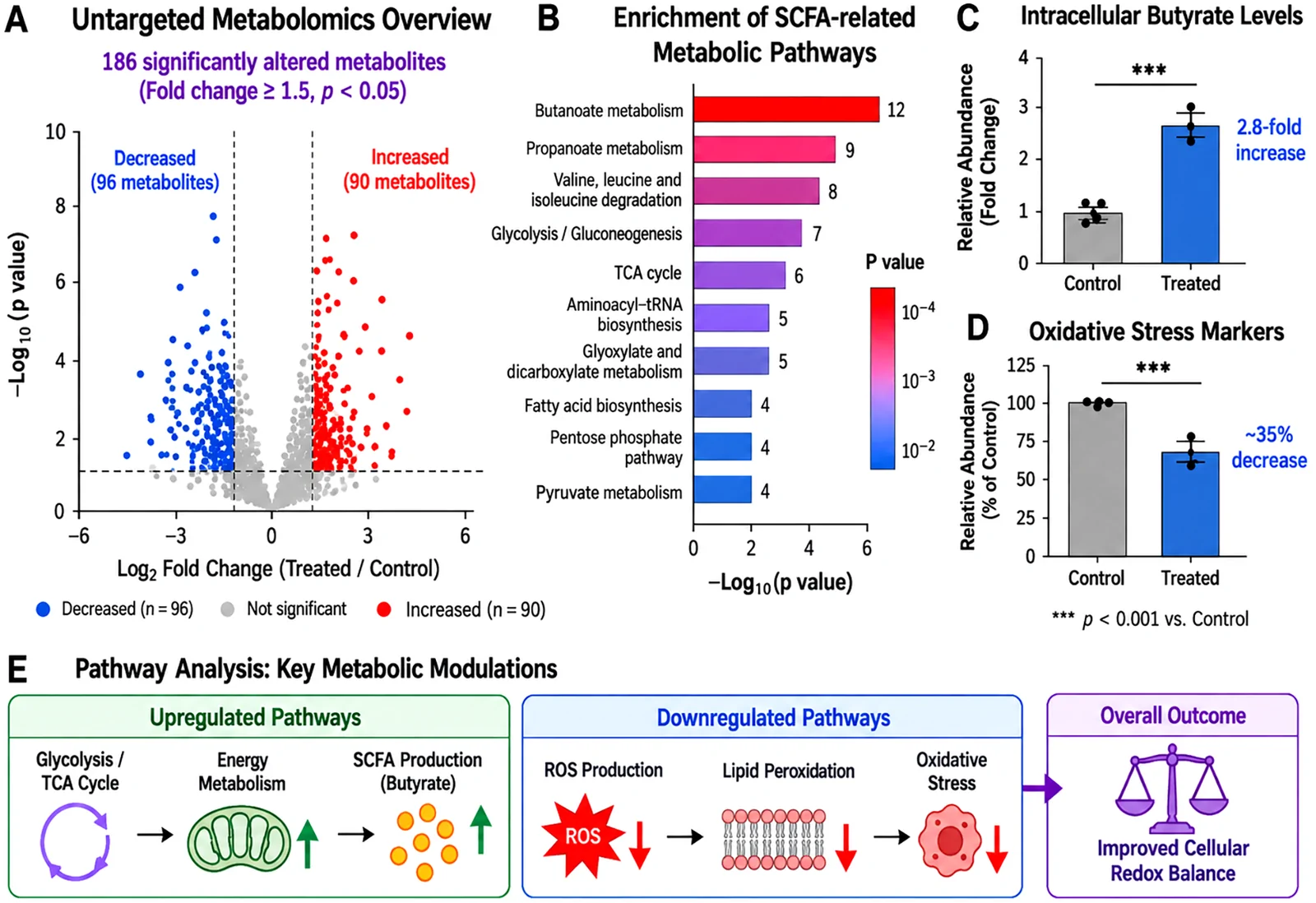
Untargeted metabolomics and pathway enrichment analysis reveal significant metabolic reprogramming following treatment. **(A)** Volcano plot showing 186 significantly altered metabolites (fold change ≥ 1.5, p < 0.05). **(B)** Pathway enrichment analysis highlighting short-chain fatty acid (SCFA)-related metabolic pathways. **(C)** Intracellular butyrate levels significantly increased (2.8-fold). **(D)** Oxidative markers were reduced by ~35 percent, suggesting reduced cellular oxidative stress. **(E)** schematic summary showing modulation of metabolism and lipid biosynthesis, which contributes to an improvement of the cellular redox state. Data presented as mean ± SEM; ***p<0.001 vs. control.

### 3.10 Microbiome analysis (16S rRNA sequencing)

16S rRNA sequencing generated high-quality microbial profiles that revealed significant alterations in community composition following probiotic metabolite treatment. At the phylum level, Firmicutes increased from 42.3% to 51.7%, while Actinobacteriota increased from 6.8% to 11.4%. In contrast, Proteobacteria decreased from 18.5% to 11.2%, and Fusobacteriota decreased from 7.6% to 4.1%. At the genus level, beneficial taxa including *Lactobacillus* and *Bifidobacterium* increased by 2.4-fold and 1.9-fold, respectively, whereas the CRC-associated genus *Fusobacterium* decreased by approximately 41%. Beta-diversity analysis demonstrated clear separation between treated and control groups, indicating treatment-induced restructuring of the microbial community. Furthermore, alpha diversity increased significantly, with the Shannon diversity index rising from 2.8 to 3.6 (p < 0.001), suggesting restoration of microbial richness and evenness (**Figure 9**).

**Figure 9:**
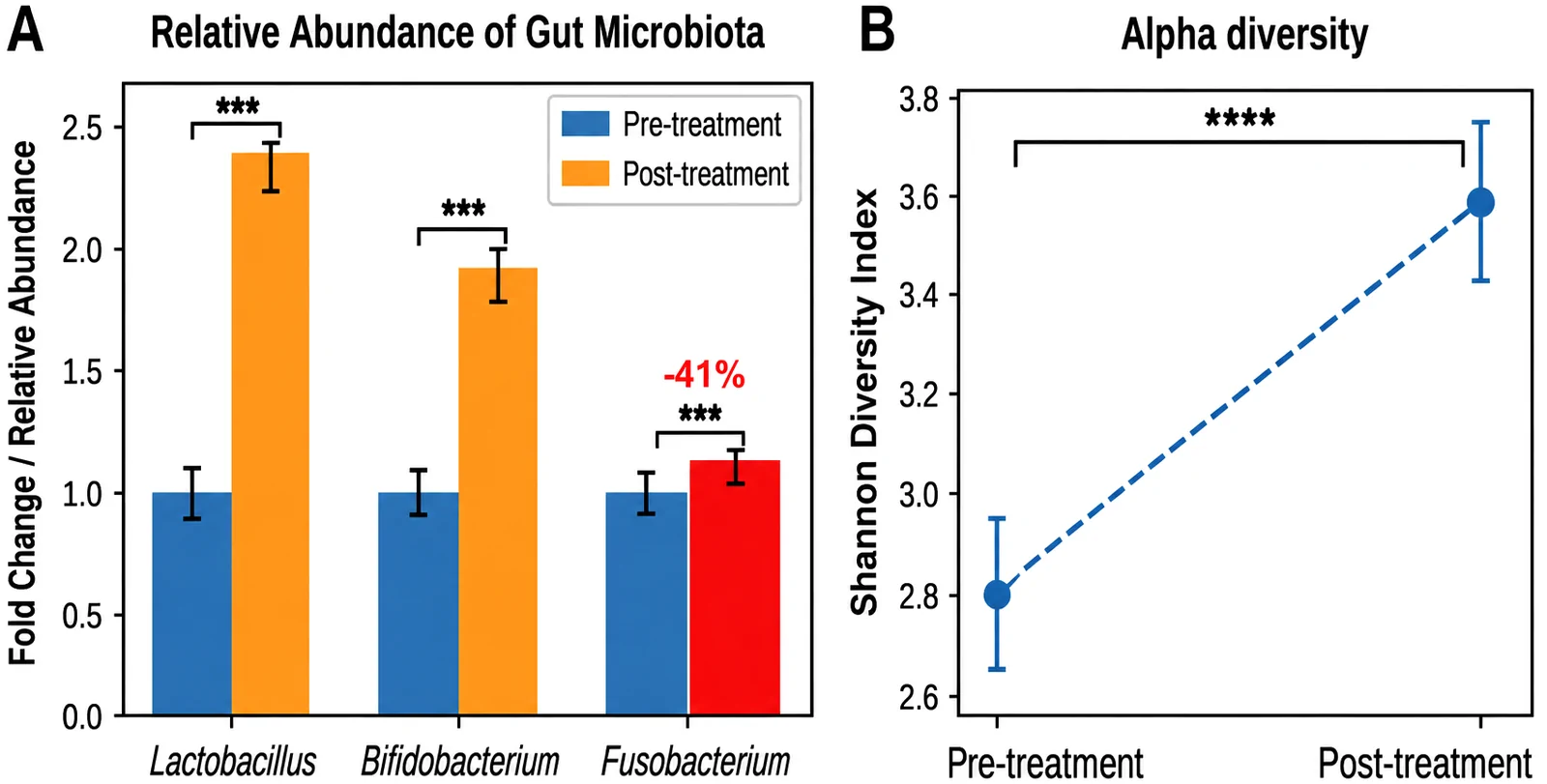
Modulation of gut microbial composition and diversity after treatment. **(a)** Relative abundance of key bacterial taxa showing an increase in *Lactobacillus* and *Bifidobacterium* and a decrease in *Fusobacilli*. **(b)** alpha diversity (Shannon index) increased significantly after treatment, suggesting an improvement in microbial diversity (***p<0.001; ****p<0.0001).

Microbiome profiling demonstrated substantial remodeling of the microbial community following probiotic metabolite treatment. At the phylum level, Firmicutes and Actinobacteriota showed increased relative abundance, whereas Proteobacteria and Fusobacteriota were significantly reduced compared with untreated controls. At the genus level, beneficial short-chain fatty acid-producing bacteria, including Lactobacillus and Bifidobacterium, were significantly enriched, while the colorectal cancer-associated genus Fusobacterium was markedly depleted. Alpha-diversity analysis revealed increased microbial richness and evenness, as evidenced by a higher Shannon diversity index. Beta-diversity analysis using Bray–Curtis distance and PCoA demonstrated clear separation between treated and control microbial communities, indicating treatment-induced restructuring of the microbiota. Collectively, these taxonomic shifts suggest restoration of a healthier microbial ecosystem characterized by enrichment of beneficial commensals and suppression of potentially pathogenic taxa associated with colorectal carcinogenesis.

### 3.11 Integrated multi-omics analysis

Integrating transcriptomic, proteomic and metabolic data has shown coordinated regulation of major hub genes and pathways. Network analysis confirmed that probiotic metabolites modulate the pathways for NF-κB, PI3K-Akt, MAPK and apoptosis by interactions with multiple targets as shown in **Table 2**. Notably, epigenetic regulation through inhibition of HDAC by butyrate has been associated with increased tumor suppressor gene expression and decreased inflammatory signaling.

**Table 2:** Integrated multi-omics analysis of probiotic metabolite-mediated pathway regulation.

Omics layer	Key findings	Affected pathways	Biological outcome
Transcriptomics	Upregulation of tumor suppressor genes	Apoptosis, NF-κB	Reduced inflammation, increased apoptosis
Proteomics	Downregulation of AKT, MAPK, inflammatory proteins	PI3K–Akt, MAPK signaling	Suppressed cell survival and proliferation
Metabolomics	Increased butyrate (SCFA), altered metabolites	Energy metabolism, lipid biosynthesis	Improved metabolic homeostasis
Epigenetics	HDAC inhibition by butyrate	Chromatin remodeling	Enhanced tumor suppressor gene expression
Network Analysis	Multi-target regulation of hub genes	NF-κB, PI3K–Akt, MAPK, apoptosis	Coordinated anti-inflammatory and anti-tumor effects

## 4 Discussion

This study provides a comprehensive systems-level understanding of the gut–cancer axis, demonstrating that probiotic-derived metabolites exert multi-target anti-cancer effects in CRC through coordinated modulation of inflammatory signaling, apoptosis, immune responses, and metabolic pathways. These findings align with recent high-impact studies emphasizing the pivotal role of gut microbiota and their metabolites in regulating tumor progression and therapeutic response in CRC (Chen et al., 2026; Dong et al., 2026; Zhao et al., 2026; Huang et al., 2026).

Gene-based hub analysis identified key regulators, including TP53, AKT1, MAPK1, PIK3CA, and NFKB1, which are central to tumor proliferation, survival, and inflammatory signaling. The observed downregulation of AKT1, MAPK1, and NFKB1, alongside upregulation of apoptotic markers such as BAX and CASP3, indicates suppression of tumor growth and activation of apoptosis. These findings are consistent with current evidence identifying dysregulation of the PI3K–Akt and MAPK pathways as major drivers of CRC progression and important therapeutic targets (Han et al., 2026; Liu et al., 2025; Hu et al., 2024).

Short-chain fatty acids (SCFAs), particularly butyrate, play a central mechanistic role in mediating anti-tumor effects. Butyrate functions as a histone deacetylase (HDAC) inhibitor, promoting epigenetic activation of tumor-suppressive pathways while suppressing pro-inflammatory signaling such as NF-κB. Emerging evidence supports the role of microbial metabolites in regulating oxidative stress and inflammation, thereby contributing to cancer prevention and progression control (Li et al., 2026; Fong et al., 2020; Yuan et al., 2026).

In addition, indole-derived metabolites contribute to immune modulation by reducing pro-inflammatory cytokine expression and enhancing anti-inflammatory responses. These observations are consistent with growing evidence that microbial metabolites shape the tumor microenvironment by influencing immune cell function and cytokine signaling, which are critical determinants of tumor progression and response to immunotherapy (Omar et al., 2026; Mattavelli et al., 2026; Wu et al., 2026).

Multivariate integration further revealed consistent modulation of key signaling pathways, including NF-κB, PI3K–Akt, and MAPK, strengthening the robustness of the findings. These pathways are closely associated with microbiota-driven signaling axes, such as Toll-like receptor-mediated inflammatory responses, which contribute to tumor initiation and progression in CRC. Recent studies highlight the central role of microbiome-driven signaling networks in integrating inflammatory and metabolic processes during tumorigenesis (Zhao et al., 2026; Zhang et al., 2026; Huang et al., 2026).

Microbiological analysis demonstrating an increase in beneficial taxa alongside a reduction in pathogenic bacteria suggests restoration of gut microbial homeostasis. Specifically, treatment promoted enrichment of Firmicutes and Actinobacteriota-associated beneficial genera, including *Lactobacillus* and *Bifidobacterium*, while reducing Proteobacteria and the CRC-associated genus *Fusobacterium*. These compositional shifts are consistent with a microbiota profile associated with reduced intestinal inflammation and improved epithelial barrier function. This shift is critical for suppressing carcinogenesis, as recent studies indicate that both gut and intratumoral microbiota composition directly influence tumor behavior and therapeutic response in CRC (Zhang et al., 2026; Chen et al., 2026; Omar et al., 2026).

*In vitro* validation across multiple CRC cell lines confirmed a significant reduction in cell viability and increased apoptosis, with enhanced effects observed upon treatment with combined metabolites. These results support the concept of synergistic interactions among microbial metabolites in modulating tumor-associated pathways. However, several limitations should be acknowledged. First, the current study was conducted primarily using *in vitro* CRC cell models and therefore lacks validation in clinical patient samples. Second, the absence of *in vivo* experiments limits the assessment of host–microbiome interactions under physiological conditions. Third, inter-individual variability in microbiome composition, metabolite production, dietary factors, and tumor heterogeneity may influence therapeutic responses and were not captured in the present experimental design. Consequently, future studies should integrate animal models and well-characterized clinical cohorts to validate the identified molecular signatures and therapeutic mechanisms.

Although the present study provides comprehensive mechanistic insights through integrated multi-omics analysis and *in vitro* validation, the findings remain limited by the absence of clinical sample verification. The transcriptomic, proteomic, metabolomic, and microbiome datasets were generated from controlled experimental systems using CRC cell lines, which may not fully capture the complexity and heterogeneity of the human tumor microenvironment. Clinical validation using patient-derived tumor tissues, adjacent normal tissues, blood samples, and fecal microbiome specimens would be necessary to confirm the translational relevance of the identified biomarkers and signaling pathways. Future investigations should incorporate multi-center patient cohorts and longitudinal clinical studies to evaluate whether probiotic metabolite-mediated modulation of the NF-κB, PI3K–Akt, MAPK, and apoptosis pathways is consistently observed in clinical CRC populations.

However, certain limitations should be acknowledged. Although sodium butyrate concentrations ranging from 1–5 mM were initially evaluated, detailed dose–response curves and IC_50_ analyses were not included in the present study. Consequently, direct quantitative comparison with previous studies remains limited. In addition, the study lacks *in vivo* and clinical validation, and physiological variability in metabolite exposure may influence therapeutic responses. Future investigations should incorporate comprehensive concentration-dependent analyses together with animal and clinical studies to further validate the observed effects.

In conclusion, this study highlights the therapeutic potential of probiotic-derived metabolites as promising adjunct therapeutic agents in CRC treatment. However, further studies involving live probiotic strains, *in vivo* models, and clinical validation are needed to determine whether these metabolite-mediated effects can be translated into probiotic-based therapeutic interventions. By targeting interconnected molecular pathways and modulating the gut microbial ecosystem, these metabolites offer a novel strategy for precision oncology. Future investigations should include validation using patient-derived clinical samples, animal models, and prospective clinical trials to confirm the biological and clinical significance of the identified multi-omics signatures. Such studies will be essential for translating probiotic metabolite-based interventions into precision oncology strategies for CRC management.

## 5 Conclusion

This study shows that metabolites derived from probiotics modulate the gut-tumor axis by modulating multi- target inflammatory, apoptotic and immune signaling pathways in CRC. Integrating multomic analysis with *in vitro* validation highlights the role of key pathways, such as NF-κB, PI3K-Akt and MAPK, in mediating these effects. These findings support the potential of probiotic-derived metabolites as adjunct therapeutic agents and provide a systems-based framework for developing microbiome-informed strategies for CRC management.

## Data Availability

Publicly available datasets were utilized in this study. Transcriptomic datasets were obtained from the Gene Expression Omnibus (GEO) under accession numbers GSE39582, GSE41258, and GSE44076, together with colorectal cancer datasets from TCGA-COAD and TCGA-READ. Public microbiome sequencing data were accessed through the Sequence Read Archive (SRA) under accession numbers PRJNA389280 and PRJNA400072. Proteomics data were obtained from the PRIDE repository (PXD002137 and PXD019463), and metabolomics data were retrieved from MetaboLights (MTBLS92). All datasets are publicly available and can be accessed through their respective repositories.
